# Whole-body MRI in paediatric oncology

**DOI:** 10.1007/s11547-015-0600-7

**Published:** 2015-12-02

**Authors:** Rutger A. J. Nievelstein, Annemieke S. Littooij

**Affiliations:** Department of Paediatric Radiology, Wilhelmina Children’s Hospital, University Medical Center Utrecht, Utrecht, The Netherlands; Imaging Division, Department of Radiology and Nuclear Medicine, University Medical Center Utrecht, P.O. Box 85500, 3508 GA Utrecht, The Netherlands

**Keywords:** MRI, Whole-body, Imaging, DWI, Paediatric oncology

## Abstract

Imaging plays a crucial role in the diagnosis and follow-up of paediatric malignancies. Until recently, computed tomography (CT) has been the imaging technique of choice in children with cancer, but nowadays there is an increasing interest in the use of functional imaging techniques like positron emission tomography and single-photon emission tomography. These later techniques are often combined with CT allowing for simultaneous acquisition of image data on the biological behaviour of tumour, as well as the anatomical localisation and extent of tumour spread. Because of the small but not negligible risk of radiation induced secondary cancers and the significantly improved overall survival rates of children with cancer, there is an increasing interest in the use of alternative imaging techniques that do not use ionising radiation. Magnetic resonance imaging (MRI) is a radiation-free imaging tool that allows for acquiring images with a high spatial resolution and excellent soft tissue contrast throughout the body. Moreover, recent technological advances have resulted in fast diagnostic sequences for whole-body MR imaging (WB-MRI), including functional techniques such as diffusion weighted imaging. In this review, the current status of the technique and major clinical applications of WB-MRI in children with cancer will be discussed.

## Introduction

The recent technical developments in computed tomography (CT), magnetic resonance imaging (MRI) and nuclear medicine have changed the role of imaging in the evaluation of children with cancer revolutionary [1, 2]. In the past, imaging techniques have been mainly used as a tool to detect tumours and to assess the extent of tumour spread before and after therapy (i.e., structural imaging). But nowadays, it has also become possible to use imaging techniques to gain information on the biological behaviour of the tumour before and during therapy (i.e., functional imaging). Until recently, CT has been the structural imaging technique of choice for staging and follow-up of malignancies. The functional imaging techniques that play a central role in paediatric cancer imaging include positron emission tomography (PET) using the radiotracer [^18^F]-2-fluoro-2-deoxy-d-glucose (FDG), and single-photon emission computed tomography (SPECT) using the radiotracer Iodine-123 metaiodobenzylguanidine (I-123 MIBG). These functional imaging techniques are usually combined with CT (i.e. PET/CT and SPECT/CT) allowing for defining anatomic localisation, lesion characterization, segmentation, and quantification of areas with abnormal tracer uptake in a single visit. A major disadvantage of all these techniques is the use of ionising radiation, which may be associated with induction of second cancers later during life. This small but not negligible health risk is of particular concern in children as their tissues are more radiosensitive than adults and they have more years ahead in which cancerous changes might occur. That is why there is an increasing interest in the use of alternative imaging techniques that do not use ionising radiation, such as ultrasonography (US) and MRI [1].

Although US is a child-friendly imaging technique that can provide real-time detailed images of most body parts, it is less useful for the evaluation of large masses, extended disease, deeper-lying tissues, and tissues located behind bones and air-containing tissues. That is why the main role of US in cancer imaging is to help ascertain the nature of palpable masses and to guide biopsy procedures. On the other hand, with MRI it is possible to acquire images with a high spatial resolution and excellent soft tissue contrast throughout the body, which makes it an ideal radiation-free tool for the detection of pathology, especially in parenchymal and bone marrow locations. Moreover, recent technological advances have resulted in fast diagnostic sequences for whole-body MR imaging (WB-MRI), including functional techniques such as diffusion weighted imaging (DWI) [3–10]. As a result, WB-MRI has become a clinically feasible imaging modality for staging and follow-up of malignancies in children [11–14]. This review will focus on the current status of the technique and major clinical applications of WB-MRI in children with cancer.

## Technique

Until now, there is no standardised technique or protocol for performing WB-MRI [3, 6]. It usually involves imaging of the entire body (from vertex to toes) but in oncology, it is often restricted to the skull or skull base to groin region in line with most hybrid imaging techniques. Most modern MRI scanners are equipped with a moving table top for sequential movement of the patient through the magnet during imaging without the need for repositioning.

Regarding the choice of coils, the use of phased-array surface coils is preferred over the use of the quadrature body coil integrated in the magnet bore, because of the better spatial resolution and signal-to-noise ratio (SNR) of the first over the latter. This is especially true when functional imaging techniques like DWI are planned to be included in the imaging protocol. The way these coils can be used for whole-body image acquisition depends on the type of MRI scanner available. One approach, the so-called “sliding table and repositioning surface coil” approach, uses tabletop spacers to place an additional table platform on the original MR table allowing manipulation of the lower part of a non-integrated phased-array surface coil without repositioning the patient [15]. However, nowadays, on several modern MRI scanners dedicated multichannel surface coil systems are available allowing for whole-body imaging without the need to reposition the coil at each station [6].

Sequences that are typically used in WB-MRI include short tau inversion recovery (STIR), T1-weighted fast spin echo (FSE, TSE), and contrast material-enhanced (CE) T1-weighted three-dimensional gradient echo (VIBE, THRIVE, LAVA) sequences. STIR is the most commonly used sequence in WB-MRI because most pathologic tissues are proton rich with prolonged T1 and T2 relaxation times resulting in high signal intensity on STIR images. Furthermore, fat suppression on STIR images is more robust and homogeneous than on T2-weighted fat-saturated images. The T1-weighted FSE sequence is especially helpful for anatomic delineation of lesions and to increase the specificity of the detection of bone marrow involvement. The addition of a 3D CE T1-weighted GRE sequence will often improve the diagnostic accuracy of lesion delineation and characterization. Furthermore, it facilitates the combination of better local tumour staging and evaluation of metastatic disease.

With the introduction of diffusion-weighted whole-body imaging with background body signal suppression (DWIBS) in 2004 by Takahara et al., it became possible to perform whole-body DWI under free breathing within a clinically acceptable examination time [5, 7, 16, 17]. To improve lesion conspicuity high b-values of up to 1000 s/mm^2^ are applied and either a STIR pre-pulse or a frequency selective (chemical shift selective, CHESS) pre-pulse is used for fat suppression to optimise background body signal suppression. Note that fat suppression is also required to avoid image degradation due to severe chemical shift when using EPI. The choice of the method of fat suppression may depend on the organ/body region under examination, although STIR usually results in the most robust fat suppression over an extended field of view, in particular in the neck/shoulder region and lower extremities. In addition, if bowel signal suppression is an important issue, STIR is the preferred method in the abdominal region as well. In case quantitative measurements of the diffusivity (apparent diffusion coefficient, ADC) in pathological tissues are required, the use of at least three b-values is recommended (including b0).

The choice of scan plane will depend on the region of interest, type of malignancy, and diagnostic information required for optimal treatment planning and follow-up. However, the coronal plane is most often acquired and displayed in whole-body imaging, in particular when STIR and T1-weighted FSE sequences are used. The 3D CE T1-weighted GRE and DWIBS sequences are usually acquired in the axial plane, but the obtained images can be easily post-processed for multiplanar reconstruction (MPR) and maximum intensity projection (MIP). MRI of the thoracic and abdominal part of the body is challenging because of movement of patient respiration and bowel peristalsis. The anatomical imaging of thorax and abdomen is usually obtained using respiratory compensation techniques, whereas the DWIBS imaging will take place under free breathing. To reduce the bowel motility, intravenously injection of an antiperistaltic agent can be considered, such as hyoscine butylbromide or glucagon. This is especially relevant if the abdomen is the site or predisposed area of the (known or suspected) primary malignancy.

In Tables 1 and 2 examples are given of two different WB-MRI protocols used in our centre for paediatric oncological indications.

**Table 1:** WB-MRI protocol—lymphoma (1.5T, Ingenia, Philips Healthcare, Best, The Netherlands)

Parameter	Pulse sequence			
	T1-weighted TSE	STIR	DWI-STIR	T2-weighted SPAIR
Repetition time (ms)	583	5231	8046	1250
Echo time (ms)	18	65	67	80
Inversion time (ms)	–	165	180	–
Receiver bandwidth (Hz)	465.9	502.7	57.3	647.0
Slice orientation	Coronal	Coronal	Axial	Axial
Slice thickness (mm)	6.0	6.0	4.0	6.0
Slice gap (mm)	1.0	1.0	0	0.7
No. of slices per station	30	30	60	36
Cranio-caudal coverage per station	265	265	240	240
Field of view (mm^2^)	530 × 265	530 × 265	450 × 365.625	450 × 366.4286
Acquisition matrix	208 × 287	336 × 133	128 × 82	280 × 202
B-values (s/mm^2^)	–	–	0, 100, 800	–
No. of signal averaged	1	1	1	1
Echo-planar imaging factor	–	–	41	–
Respiratory motion compensation technique	Breath hold in thorax and abdomen	Breath hold in thorax and abdomen	Free breathing	Free breathing
Acquired voxel size (mm^3^)	1.27 × 1.85 × 6.00	1.58 × 1.98 × 6.00	3.52 × 4.46 × 4.00	1.61 × 1.81 × 6.00
Reconstructed voxel size (mm^3^)	1.04 × 1.04 × 6.00	1.04 × 1.04 × 6.00	2.01 × 1.99 × 4.00	0.94 × 0.93 × 6.00
Effective scan time per station	54 s	1 min 45 s	4 min	45 s
Total number of stations	5	5	4	4
Total effective scan time	4 min 30 s	8 min 45 s	16 min	3 min

**Table 2:** WB-MRI protocol—neuroblastoma (1.5T, Ingenia, Philips Healthcare, Best, The Netherlands)

Parameter	Pulse sequence					
	3D T2W TSE	T1 THRIVE	DWI	T1 TSE (spine)	STIR (spine)	T1 THRIVE Gd
Repetition time (ms)	454	5.5	1341	550	2000	5.5
Echo time (ms)	90	2.7	73	8.0	60	2.7
Inversion time (ms)	–	–	–	–	180	–
Receiver bandwidth (Hz)	570.1	309.6	47.9	206.1	383.0	309.6
Slice orientation	Coronal	Axial	Axial	Sagittal	Sagittal	Axial
Slice thickness (mm)			4.0	3.0	3.0	
Slice gap (mm)			0	0.3	0.3	
No. of slices per station	139	85	26	15	15	85
Cranio-caudal coverage per station	400	127.5	130	449.1228	448	127.5
Field of view (mm^2^)	400 × 354.023	250 × 189.2759	250 × 250	449.1228 × 160	448 × 160	250 × 189.2759
Acquisition matrix	348 × 308	232 × 185	88 × 70	228 × 494	160 × 381	232 × 185
B-values (s/mm^2^)	–	–	0, 100, 1000	–	–	–
No. of signal averaged	1	4	1	2	2	4
Echo-planar imaging factor	–	–	35	–	–	–
Respiratory motion compensation technique	Respiratory triggered	Free breathing	Respiratory triggered	Free breathing	Free breathing	Free breathing
Acquired voxel size (mm^3^)	1.15 × 1.15 × 1.15	1.08 × 1.07 × 3.00	2.84 × 3.57 × 5.00	0.70 × 0.91 × 3.00	1.00 × 1.17 × 3.00	1.08 × 1.07 × 3.00
Reconstructed voxel size (mm^3^)	0.83 × 0.83 × 1.15	0.74 × 0.74 × 1.50	1.74 × 1.74 × 5.00	0.47 × 0.47 × 3.00	0.70 × 0.70 × 3.00	0.74 × 0.74 × 1.50
Effective scan time per station	4 min 09 s	2 min	1 min 48 s	5 min 10 s	4 min	2 min
Total number of stations	1	3	3	1	1	3
Total effective scan time	4 min 09 s	6 min	5 min 24 s	5 min 10 s	4 min	6 min

## Clinical applications

### Bone marrow imaging

Bone marrow has three primary components: osseous matrix, red marrow and yellow marrow. The osseous components provide supporting framework to the red and yellow marrow elements. The red marrow is composed of haematopoietic cells that produce the peripheral blood precursors, whereas in yellow marrow fat cells make up the vast majority. The distribution of haematopoietic marrow and its cellular content vary with age. At birth, marrow is entirely haematopoietic. Shortly after birth, the transition to yellow bone marrow occurs at an orderly and predictable sequence, which begins in the peripheral bones and progresses in a symmetrical manner to the central skeleton. Within the individual long bones, the marrow conversion occurs first in the diaphysis that progresses to the metaphysis. In the first decade of life the vertebral marrow is predominantly haematopoietic except for some yellow bone marrow around the central vertebral vein [18].

**Fig. 1:**
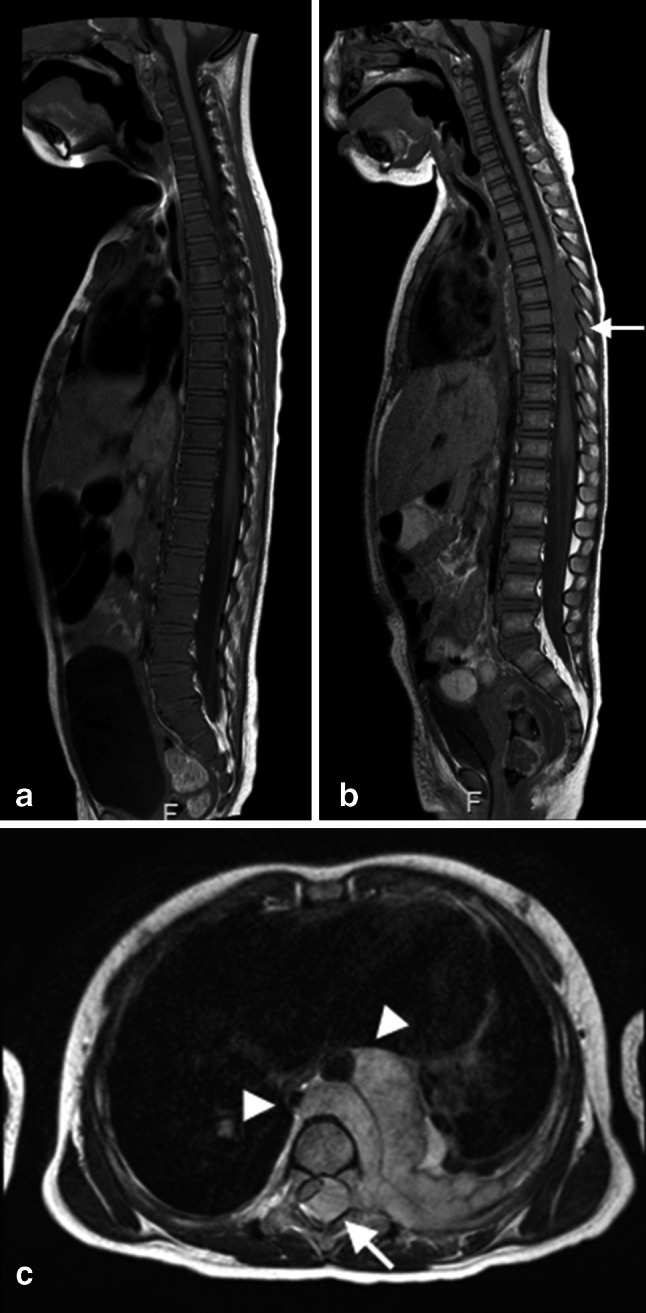
Sagittal T1 spin echo (SE) image in two patients diagnosed with neuroblastoma. **a** The image of a 3-year-old girl with proven bone marrow involvement illustrate the diffuse low signal intensity of the bone marrow of the spine compared to the intervertebral disk that is suggestive for diffuse bone marrow infiltration, whereas normal hematopoietic bone marrow is slightly hyperintense to the intervertebral discs of the spine at T1 weighted imaging. **b** The image of a 2-year-old girl with thoracic neuroblastoma shows the appearance of not-involved bone marrow within the vertebral bodies. **c** Transverse T2-W image demonstrates the intraspinal extension with spinal cord compression (*arrow*, see also the *arrow* in **b**) as well as the vascular encasement and the lifting of the aorta (*arrowheads*) that is considered one of the key imaging features of neuroblastoma

MRI is a sensitive method for assessing bone marrow; however, it lacks specificity. Normal bone marrow is rich in fat and water that together contribute to the signal seen on MRI. Red marrow contains approximately 40 % fat and 40 % water whereas yellow marrow is composed of approximately 80 % fat and 15 % water. TSE sequences allow superb differentiation between red and yellow bone marrow as the signal characteristics of fatty and hematopoietic marrow are different. However, the T1-weighted sequence will detect all the fat in the marrow inclusive the 40 % fat that is present in the hematopoietic marrow. Therefore, hematopoietic marrow is slightly hyperintense to normal muscles and the intervertebral discs of the spine (Fig. 1b). On water sensitive images (i.e. STIR) hematopoietic marrow is of higher signal intensity than fatty marrow [3, 9].

Malignant infiltration of the bone marrow is associated with free water and oedema in addition to replacement of the fatty content of normal marrow. Therefore, pathological bone marrow is of low T1 and high T2 signal (Fig. 1a). Especially in young patients it can be challenging to detect bone marrow disease, because the high cellularity of normal red marrow can be misdiagnosed as diffuse bone marrow infiltration or mask tumour deposits. Chemical shift imaging (oppose phase imaging) could be used as a complementary tool in order to differentiate between malignant bone marrow infiltration and normal red marrow by detecting small quantities of fat. Previous studies have shown that chemical shift imaging allows distinction between benign and malignant causes in adult patients [19, 20].

DWI in assessment of bone marrow disease is, especially in young patients, of limited value, as the high cellular haematopoietic marrow will exhibit impeded diffusion. Ording-Müller et al. [21] demonstrated that areas of restricted diffusion in the pelvic skeleton and lumbar spine are a normal finding in 48 % of healthy children. Therefore, DWI, if used in isolation, could increase the false-positive rate of bone marrow pathology.

Meyer et al. [22] tried to identify the best MRI sequence or image criteria for the diagnosis of bone marrow metastases in children with neuroblastoma. They found that homogenous low T1 signal had the highest sensitivity (88 %), whereas a heterogeneous pattern on the post-gadolinium was highly specific (97 %), but relatively insensitive (65 %) for detecting metastasis.

### Malignant lymphoma

Lymphoma [Hodgkin Disease (HD) and non-Hodgkin lymphoma (NHL)] is the third most common form of malignancy in children, after leukaemia and brain tumours [23]. NHL is most frequent in children younger than 15 years whereas HD is predominantly diagnosed in teenagers. A major challenge in treating paediatric lymphoma is to optimise up-front treatment to prevent disease relapse, while minimising late therapy-related side effects. Accurate assessment of disease extent at diagnosis and response to treatment are therefore essential. Paediatric lymphomas are staged using the modified Ann Arbor and Murphy classifications for HD and NHL, respectively [24, 25].

**Fig. 2:**
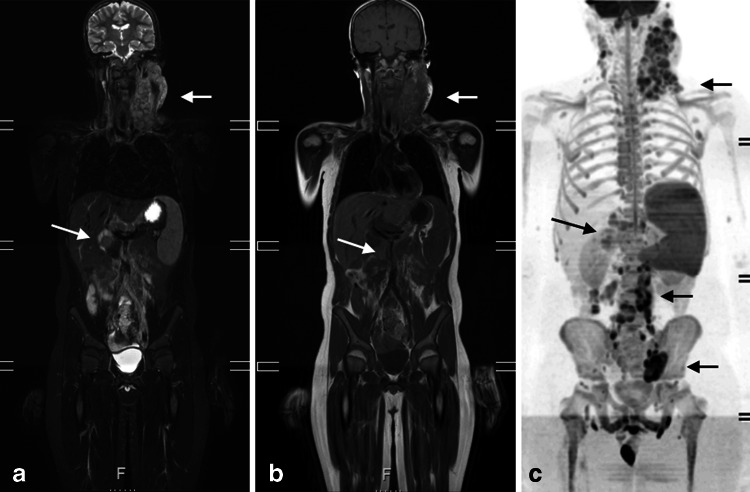
Coronal whole-body short tau inversion-recovery (**a**), T1-W (**b**) and maximum intensity projection greyscale inverted diffusion-weighted (**c**) images of a 12-year-old boy with anaplastic large cell lymphoma illustrate nodal involvement at both sides of the diagram (*arrows*) and an enlarged spleen (splenic index 763 cm^3^) indicating splenic involvement diagnosed with whole-body MRI. The paraaortal and left parailiacal involvement as illustrated at the diffusion-weighted image is not seen at the coronal STIR and T1-W images as this area was not included in these slices

Current guidelines encourage the use of FDG-PET/CT in staging and response assessment of FDG-avid lymphomas [24]. WB-MRI could be a good radiation-free alternative for staging and follow-up. Several studies have shown that WB-MRI is feasible, even in children (Fig. 2) [26–28]. Punwani et al. [27] reported very good agreement between WB-MRI compared to an FDG-PET/CT reference standard for nodal and extranodal disease, despite only using STIR for whole-body imaging.

There is an increased interest for the use of DWI in lymphoma due to the clear visualisation of the lymphoid tissue with DWI that is thought to increase the detection rate while decrease reading time. Lin et al. [29] showed an excellent agreement between WB-DWI and FDG-PET/CT in 15 adult patients with diffuse large B cell lymphomas. However, in other studies the additional value of DWI to conventional sequences could not be demonstrated [28, 30]. This could be related to the fact that both benign and malignant nodes demonstrate impeded diffusion. Also, there are no validated ADC values yet for discriminating involved from not involved sites [30]. Therefore, the detection of lymph nodes in WB-DWI is still based on size criteria. An important issue with DWI is that several normal extranodal structures (including brain, salivary glands, Waldeyer ring, thymus, spleen, gallbladder, adrenal glands, prostate, testes, penis, endometrium, ovaries, spinal cord, peripheral nerves and bone marrow) may demonstrate impeded diffusion. Consequently, pathology in any of these areas may be obscured.

Klenk et al. [14] prospectively compared Ferumoxytol-enhanced whole-body diffusion-weighted MRI to FDG-PET/CT in 22 children and young adults for staging lymphoma and sarcoma. This iron oxide contrast is thought to overcome the limitations of conventional WB-MRI regarding detecting bone marrow and splenic involvement. This better detection of lymphomatous involvement in the reticuloendothelial system (RES) depends on the uptake of USPIO through macrophages in the non-involved RES and the non-uptake by metastatic tumour deposits.

Assessment of response to therapy is important for determining treatment effectiveness and predicting clinical outcome. The concept of early response assessment with FDG-PET/CT in lymphoma has received considerable attention last years, however it is still not officially recommended outside clinical trails [24]. The role of WB-MRI in response assessment in children with lymphoma is still under investigation. Mayerhoefer et al. [31] recently published their results of their prospective study in 64 adult lymphoma patients that showed that WB-MRI with DWI could serve as a feasible alternative for FDG-PET/CT during follow-up and treatment response assessment. Several, mostly pilot, studies compared the quantitative data from FDG-PET/CT (SUV) with DWI (ADC values) for interim response assessment with inconclusive results. They reported presence or absence of an inverse correlation between ADC and SUV [32–35].

Although FDG-PET/CT is the imaging technique of choice for treatment response assessment, the value of interim and end-of-treatment FDG-PET/CT in predicting outcome was demonstrated to be unsatisfactory by recent meta-analyses [36, 37]. Therefore, prospective studies that correlate DWI results with patient outcome measures instead of FDG-PET/CT will provide reliable evidence.

### Histiocytosis

Langerhans cell histiocytosis (LCH) is a rare disease that is characterised by lesions that include CD207+ dendritic cells with phenotypic similarity to epidermal Langerhans cells on a background of inflammatory cells [38]. The incidence of LCH is approximately 5:1,000,000 children younger than 15 years of age with an equal distribution among boys and girls. The median age of presentation is 30 months. LCH can involve almost any organ, and the most common presentation includes skin rashes and/or painful bone lesions. Less frequently, children present with diabetes insipidus due to pituitary involvement or back pain caused by vertebra plana. For prognostic purposes, patients are usually divided into a clinical “high-risk” versus “low-risk” group, based on the presence or absence of liver, spleen and/or bone marrow involvement. “High-risk” LCH patients have a >85 % long-term survival rate, whereas the survival rate of “low-risk” LCH patients approaches 100 %.

**Fig. 3:**
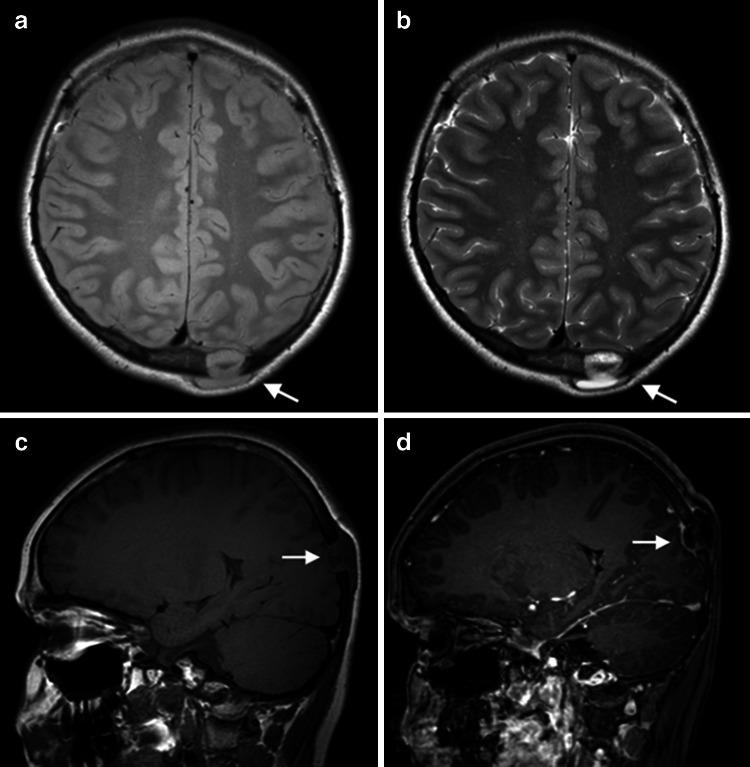
14-year-old male with Langerhans cell histiocytosis (LCH) presenting with a painless swelling on the skull. Axial proton density weighted image (**a**), T2-weighted image (**b**), and sagittal T1-weighted images before (**c**) and after the administration of intravenous contrast material (**d**). The lesion shows almost homogeneous T2 hyperintensity and slightly heterogeneous T1 intermediate signal intensity (*arrow* in **a**–**c**). After contrast material administration there is a predominantly peripheral enhancement pattern, less often seen in LCH lesions (*arrow* in **d**)

**Fig. 4:**
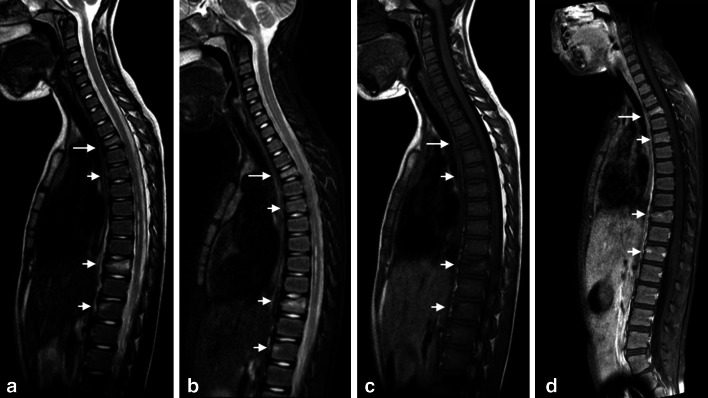
A 7-year-old girl with Langerhans cell histiocytosis (LCH) presenting with back pain. Sagittal T2-weighted (**a**), STIR (**b**), T1-weighted (**c**), and contrast-enhanced fat saturated T1-weighted (**d**) images of the spine showing involvement of multiple vertebral bodies (*small arrows*) as well as a “vertebra plana” at the level of Th3 (*large arrow*). The lesions are best seen on the precontrast T1-weighted and T2-weighted images

Adequate staging at diagnosis is essential, not only for determining prognosis, but also for choice of therapy, as lesions in more than one site usually impact the need for systemic chemotherapy. The standard approach to staging usually consists of a combination of laboratory tests and imaging [38, 39]. Traditionally, the imaging evaluation of patients with LCH includes chest radiography and a complete skeletal survey. Nowadays an abdominal ultrasound is usually added to rule out/demonstrate intra-abdominal organ involvement. Skeletal involvement is the most common radiographic abnormality in LCH. Primary sites of bony involvement are the skull, ribs, spine (vertebra plana), pelvis and scapula. The long bones are less frequently involved, with the femora as the most commonly involved long bone. Extension of the primary bony lesion in the surrounding soft tissues and epidural space can be seen, especially in the skull, ribs and spine. Bone scintigraphy has been used for the evaluation of LCH, and although lesion detection is usually higher when compared to the skeletal survey, the scintigraphic appearance of LCH may vary especially when the lesions are small or fail to incite a significant osteoblastic response [39, 40]. The use of 18F-FDG PET for the evaluation of paediatric LCH has been reported, and several recent studies have shown high sensitivity and specificity of this imaging technique superior to the skeletal survey and bone scintigraphy [41]. In addition, 18F-FDG PET allows for the identification of extra-osseous localisations of LCH and shows lesion response to therapy earlier than conventional radiography and CT. However, a recent study by Mueller et al. [41] also showed that the overall sensitivity of 18F-FDG PET was lower than WB-MRI, especially for small bony infiltrates (mean diameter 12 mm), and central nervous system involvement. As stated before, WB-MRI is very well suited for the evaluation of bone marrow involvement, accompanying soft-tissue masses, and other extra-osseous manifestations. In LCH, most bony lesions will show intermediate–hypointense signal on T1-weighted images and hyperintense signal on T2-weighted and STIR images (Figs. 3, 4) [42, 43]. On postcontrast T1-weighted images the LCH lesions usually show marked enhancement although predominantly peripheral enhancement can be seen. In addition, early-stage lesions will show oedema in the adjacent bone marrow, periosteum and soft tissues. Direct extension in the adjacent soft tissues and epidural space is very well appreciated with MRI. The same holds true for the extra-osseous organ involvement. During treatment, healing lesions will show decrease in signal intensity on STIR imaging. Unfortunately, large prospective studies on the diagnostic accuracy of WB-MRI in paediatric LCH are missing. Goo et al. [42] compared the use of WB-MRI to conventional radiography and bone scintigraphy in 9 children with LCH. Additional skeletal lesions were identified by WB-MRI in 3 out of 8 patients compared with plain radiography (38 %), and in 2 out of 8 patients compared with scintigraphy (25 %). Furthermore, WB-MRI detected extra-osseous lesions in 5 of nine patients exclusively (56 %). Steinborn et al. [43] did investigate the use WB-MRI in 6 children with LCH at diagnosis and during follow up. In comparison with the skeletal survey, WB-MRI did detect additional lesions in two patients, which resulted in a change of therapy in both children. On the other hand, Mueller et al. [41] did show that, although WB-MRI was more sensitive for the detection of LCH lesions when compared 18F-FDG-PET, specificity was significantly lower (67 and 76 % for PET vs. 81 and 47 % for WB-MRI). The lower specificity of WB-MRI was mainly due to the detection of false-positive bone lesions. Furthermore, residual T2 hyperintensity and contrast-enhancement after treatment on WB-MRI resulted in false-positive findings during follow-up. Interestingly, a combined analysis of PET and MRI decreased the number of false-negative findings at primary staging, whereas no advantage over PET alone was seen in terms of false-positive or false-negative results during follow-up.

### Neuroblastoma

Neuroblastoma (NBL) is the most common solid extra-cranial tumour in children and infants. It represents approximately 6 % of all cases of childhood cancer and accounts for 15 % of cancer deaths in children [23]. NBL is an embryonic tumour arising from primordial neural crest cells that are the precursors of the sympathetic nervous system. The most common site of the primary tumour is within the abdomen (the adrenal medulla in 35 %), but it can occur anywhere along the sympathetic chain from neck to pelvis [44]. Nearly 70 % of children with NBL will have metastatic disease at diagnosis (cortical bone, bone marrow, lymph nodes, liver and skin). NBLs have a variable prognosis. Some tumours behave aggressively, while others, often in the younger age group, may spontaneously regress [44].

Ninety percentage of patients are diagnosed before the age of 6, with a peak incidence around 2–3 years [44]. Clinical presentation varies with the size, spread and location of the tumour in often an unwell child. In the abdomen it can cause abdominal distension, sometimes nausea or pain. Unexplained fevers, bone pain or limping is caused by bone or bone marrow involvement. If the tumour is compressing the spinal cord it causes muscle weakness or problems with urinating or defecation. Some tumours produce hormones that can cause high blood pressure, increased heart rate, flushing or diarrhoea.

Since 1986, the International Neuroblastoma Staging System (INSS) has been used as a post-surgical staging system. In 2009 a new staging system was published [the International Neuroblastoma Risk Group Staging System (INRGSS)], shifting focus to pre-treatment staging with identification of imaging defined risk factors (IDRF) [45]. These IDRFs describe the relationship between the tumour and adjacent structures that ideally should not be injured during surgery (i.e., major vascular encasement, airway compression or CNS infiltration).

**Fig. 5:**
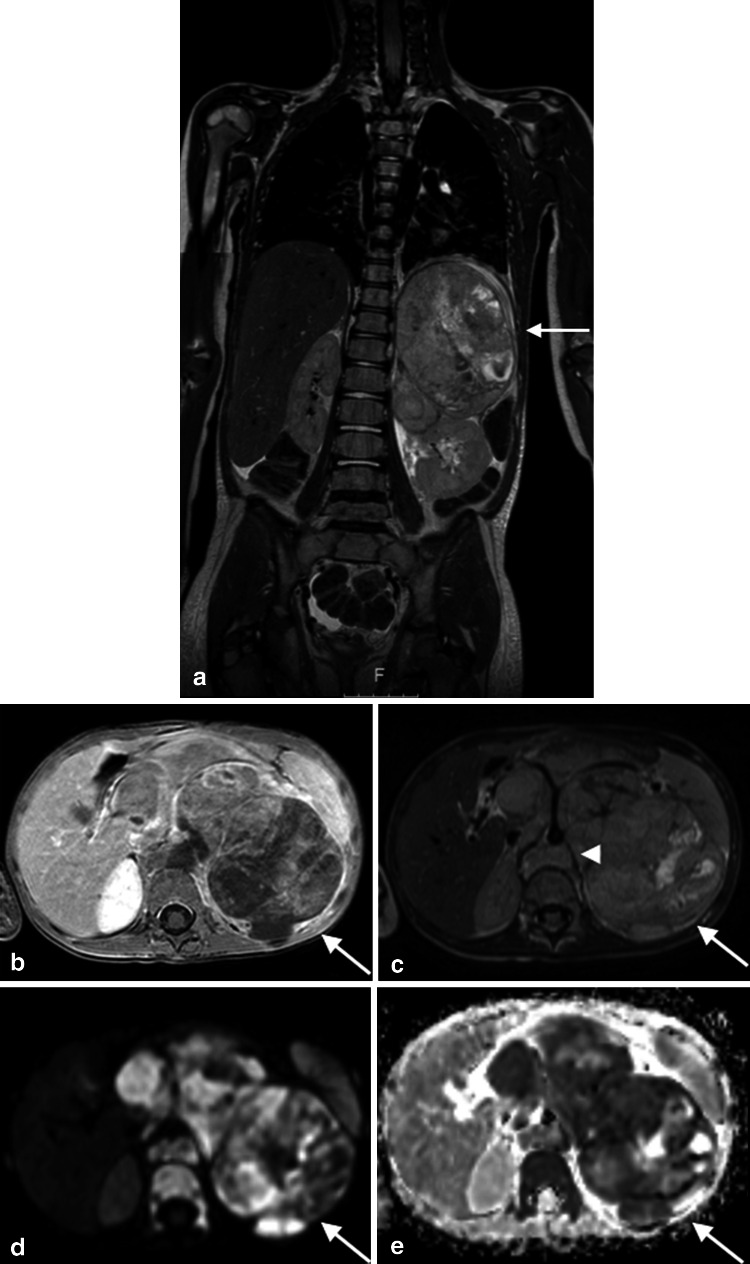
A 4-year-old girl diagnosed with stage IV neuroblastoma. Coronal acquired 3D T2-weighted image (**a**) with axial reconstruction (**c**) demonstrates the primary tumour arising from the left adrenal gland (*arrows*) with compression upon the left kidney, vascular encasement and lifting of the aorta (*arrowhead*). The post-contrast T1-weighted image illustrates heterogeneous enhancement (**b**). There is diffusion restriction seen as high signal at the b1000 image (**d**) with corresponding low signal at the ADC map (**e**)

The combination of nuclear medicine and radiological examinations are crucial for accurate staging, defining resectability and follow-up during treatment. Traditionally, CT, I-123 MIBG scintigraphy and bone marrow biopsies were used to evaluate the local and distant extend of disease. However, MRI is increasingly used for anatomical imaging (Fig. 5) [44]. The impact of WB-MRI in neuroblastoma has not been thoroughly evaluated. Pluger et al. [46] retrospectively reviewed 50 MRI and I-123 MIBG examinations in 28 patients for the assessment of neuroblastoma lesions at presentation and follow-up. They concluded that integrated imaging with I-123 MIBG scintigraphy and MRI increased the diagnostic accuracy. Gahr et al. [47] studied the role of DWI in differentiating neuroblastoma and ganglioneuroblastoma/ganglioneuroma in 15 patients with 16 histologically classified tumours. They found that there was a significant difference in ADC between neuroblastoma and ganglioneuroblastoma/ganglioneuroma. However, the inter- and intraobserver variability of their applied method was not tested. Besides, there was a considerable overlap in ADC values between these groups.

### Cancer predisposition syndromes

WB-MRI is a promising imaging tool in the evaluation of genetic cancer predisposition syndromes (CPS), especially because of its lack of ionising radiation [48, 49]. Children with CPS (including neurofibromatosis type 1, Beckwith-Wiedemann, multiple endocrine neoplasia, Li-Fraumeni, Von Hippel-Lindau, and rhabdoid tumour syndrome) are at a significantly increased risk of developing cancer, but when and in which organs tumours will develop is difficult to predict. Therefore, screening on a regularly basis is usually recommended in these children, the frequency of which depends on the specific syndrome and risk stratification. Several publications already did mention the use of WB-MRI as a screenings method in CPS, but studies on the performance of this technique in children is scarce. Friedman et al. [48] retrospectively investigated the role of WB-MRI as a screening tool in follow-up of children and adolescents with hereditary retinoblastoma. WB-MRI detected suspicious lesions in 5 of 25 patients during follow-up of which only 2 appeared to be malignant (osteosarcoma). One additional patient was diagnosed with osteosarcoma 3 months after a normal WB-MRI. The sensitivity of WB-MRI to detect subsequent malignant lesions in this specific patient group was 66.7 % and the specificity 92.1 %. Another recent (retrospective) study, which included 50 WB-MRI examinations in 24 children with genetic CPS, did show that WB-MRI is a valuable screening tool with a high sensitivity of 100 % (95 % CI 6–100 %), specificity of 94 % (82–98 %), and negative predictive value of 94 % (90–100 %) [49]. In nine of the 50 WB-MRI examinations a suspicious lesion was found; 2 high-risk, 2 moderate-risk, and 5 low-risk lesions. Of the 4 high- to moderate-risk lesions, only one lesion appeared to be malignant resulting in a positive predictive value of only 25 % (95 % CI 1–78 %). The other lesions and all low-risk lesions had a benign origin. Of interest, this study did also show that incidental findings were detected in 23 of 24 patients, most of which did not require imaging follow-up. Finally, all abnormalities were best detected on the (fluid-sensitive) STIR images. The role of DWI has not been investigated. Both studies conclude that, although WB-MRI can be regarded as a valuable screening tool, larger cohort studies are needed to validate its role and cost-effectiveness in this specific group of patients. Furthermore, Anupindi et al. [49] recommend that interpretation of these studies should be reserved to radiologists that are familiar with WB-MRI to appropriately risk stratify abnormalities and minimise unnecessary interventions.

### Future perspectives

In children, the choice of imaging modality is driven mainly by reducing the radiation dose as much as possible. This also accounts for children with cancer, as their overall survival rates have increased significantly over the past decades. This explains the increasing interest for the use of (whole-body) MRI in the diagnostic work-up and follow-up of children with cancer. As illustrated in this review, WB-MRI is already widely used in paediatric oncology, despite the fact that validation of the technique is mainly based on retrospective data in relatively small patient groups. There is a strong need for large prospective cohort studies to better validate the role and cost-effectiveness of WB-MRI in children with cancer, both for diagnosis as well as therapy response assessment. However, due to the comparatively low incidence of most paediatric malignancies, most institutions will not have sufficient patient volume to perform clinical trials that will result in definitive and statistically significant data. This obviates the need for increasing international multi-institutional collaboration and set up of multicentre clinical trials [50].

In oncology, the role of imaging is moving from structural (anatomic) imaging to combined structural and functional (molecular) imaging, enabling better tumour characterization, prognostication, response assessment, and prediction of outcome of therapy. That is why research on WB-MRI in children not only should focus on further optimization of anatomic sequences (including reduction of scan duration) but also on the introduction and validation of new functional techniques. DWI is already widely used, but the value of DWI and ADC measurements in diagnosis and follow up still has to be validated. Other functional techniques focus on tumour vascularisation, such as dynamic contrast-enhanced MRI (DCE-MRI) and arterial spin labelling (ASL). A major advantage of ASL over DCE-MRI is that it does not need intravenous contrast material injection, which makes it an ideal tool for children. Magnetic resonance spectroscopy (MRS) allows for a non-invasive separation of the MRI signal from a given tissue into its different chemical components, which may improve lesion characterization and prediction of clinical outcome. Although ASL and MRS are already established techniques in neuroradiology, their possible role in (paediatric) malignancies outside the brain has to be validated yet. In this scope, the recent development of integrated PET/MRI systems is very interesting, combining the superior structural imaging of MRI with the functional (molecular) information of both imaging techniques while decreasing the radiation dose. Although the first publications in children show promising results, it currently remains a technical challenge to construct and use these hybrid systems [51–53].

## Conclusion

WB-MRI is a very promising and already widely used imaging technique in paediatric oncology. This is mainly due to the lack of ionising radiation, its superior tissue contrast and potential to non-invasively generate functional information on tumour biology. It provides complementary information to the increasingly used molecular imaging techniques like PET/CT and SPECT/CT. However, there is still a strong need for prospective large cohort studies to better validate the role and cost-effectiveness of WB-MRI in paediatric oncology.
